# A rare germline mutation reverses the suppressive effect of *GPC5* thereby promoting lung adenocarcinoma development and tumorigenesis

**DOI:** 10.3389/fgene.2025.1582504

**Published:** 2025-04-25

**Authors:** Zhifa Zheng, Lina Zhao, Sen Zhao, Zhihong Wu, Nan Wu

**Affiliations:** ^1^ Department of Orthopedic Surgery, Peking Union Medical College Hospital, Chinese Academy of Medical Sciences and Peking Union Medical College, Beijing, China; ^2^ Medical Research Center, Peking Union Medical College Hospital, Chinese Academy of Medical Sciences and Peking Union Medical College, Beijing, China; ^3^ Beijing Key Laboratory of Big Data Innovation and Application for Skeletal Health Medical Care, Beijing, China; ^4^ Key Laboratory of Big Data for Spinal Deformities, Chinese Academy of Medical Sciences, Beijing, China; ^5^ Department of Molecular and Human Genetics, Baylor College of Medicine, Houston, TX, United States

**Keywords:** lung adenocarcinoma (LUAD), familial lung cancer, glypican-5 (GPC5), germline mutation, epithelial-mesenchymal transition (EMT)

## Abstract

**Background and Objective:**

Glypican-5 (GPC5) has been well-characterized as a tumor suppressor in lung adenocarcinoma (LUAD); however, the functional implications of its germline mutations in cancer pathogenesis remain largely unexplored. In this study, we identified and characterized a pathogenic GPC5 variant (c.776C>T, p.Pro259Leu) within a Chinese LUAD pedigree, systematically investigating its oncogenic mechanisms through comprehensive molecular and cellular analyses.

**Methods:**

Our investigation employed a multifaceted approach beginning with the recruitment of a LUAD-affected family cohort (n=4 patients, 1 healthy control), followed by exome sequencing of matched blood and FFPE tumor samples. Through rigorous rare variant analysis, we prioritized the GPC5 c.776C>T variant, subsequently validating its pathogenicity via integrated computational modeling and immunohistochemical profiling. Mechanistic studies in A549 and H2009 LUAD cell lines encompassed: (1) comprehensive proliferation and apoptosis assessment using CCK-8, colony formation, EdU incorporation, and flow cytometry; (2) migration and invasion evaluation through Transwell and wound healing assays; (3) EMT/Wnt pathway interrogation via Western blot analysis of E-cadherin, N-cadherin, Vimentin, and β-catenin expression patterns; and (4) definitive functional validation through GPC5 overexpression and knockdown experiments.

**Results:**

Genetic analysis revealed the GPC5 c.776C>T variant exhibited complete cosegregation with LUAD phenotype in the pedigree while being absent in control populations (gnomAD frequency: 0.000003989), accompanied by significantly reduced GPC5 expression in tumor tissues. Functional characterization demonstrated that compared to wild-type, the mutant variant conferred aggressive oncogenic properties: significantly enhanced proliferative capacity, impaired apoptosis induction, and markedly increased migratory potential. Molecular analyses revealed the mutant promoted EMT activation through nuclear β-catenin accumulation and subsequent upregulation of mesenchymal markers. Crucially, siRNA-mediated GPC5 knockdown phenocopied these oncogenic effects, providing definitive evidence of its tumor-suppressive function.

**Discussion:**

Our findings establish that the GPC5 c.776C>T mutation drives LUAD progression through a novel molecular mechanism involving impaired β-catenin degradation, subsequent nuclear translocation, and consequent EMT activation. These results position GPC5 as a critical nodal regulator of Wnt/β-catenin signaling in LUAD pathogenesis and suggest its germline mutations may serve as valuable biomarkers for hereditary LUAD risk assessment. Therapeutically, these findings highlight the potential utility of Wnt pathway inhibitors in managing GPC5-mutant LUAD cases, while also providing a molecular framework for future investigations into glypican family members in cancer biology.

## Introduction

Lung adenocarcinoma (LUAD) emerges as the predominant subtype of primary lung cancer, accounting for roughly 50% of all lung cancer cases (Luo et al., 2025; Sung et al., 2021). The principal risk factors for LUAD encompass exposure to smoke, a history of chronic obstructive pulmonary disease (COPD), occupational hazards, a family history of lung cancer, and genetic predispositions. A significant genetic component is evident in LUAD, with approximately 8% of patients having family histories of the disease, referred to as familial lung cancer (FLC). FLC is more complex compared to other hereditary cancers, potentially owing to shared environmental influences or genetic factors among relatives (Hemminki et al., 2025). The contribution of genetic factors to the development of lung cancer remains unclear, as environmental factors such as smoking, air pollution, and coal burning exert a more pronounced influence (He et al., 2025). The genetic predisposition to LUAD is categorized into two main types: individual mutations in critical genes and single nucleotide polymorphisms (SNPs). Pathogenic variants in EGFR (V843I, T790M, R776H), ATR (T2556S), and NBN (Q494Tfs*10) have been linked to dominantly inherited forms of LUAD (Alsaddah et al., 2023; Eiamart et al., 2025; Gabriel et al., 2024; Bao et al., 2022; Xu et al., 2022). Moreover, a novel germline mutation in the transmembrane domain of Human Epidermal Growth Factor Receptor 2 (*HER2*) in LUAD has been identified recently (Tan et al., 2022).

Beyond rare Mendelian variants, single nucleotide polymorphisms significantly augment the genetic risk of LUAD. A genome-wide analysis revealed that *rs2352028* at 13q31.3 increases the lung cancer risk by 1.46-fold in nonsmokers (Zhang et al., 2021). Notably, *rs2352028* is associated with lower expression of glypican-5 (*GPC5*), a known lung cancer tumor suppressor (Landi et al., 2010). The *GPC5* gene, situated on chromosome 13q31.3, comprises 8 exons and encodes a protein of 572 amino acids, spanning a genomic region of 1.47 megabases. *GPC5 rs2352028* C>T may exert a protective effect on lung cancer patients, and *GPC5 rs2352028* may represent a potential genetic marker for the prognosis of lung cancer. The functional relevance of Glypican-5 (the gene product of *GPC5*) in the regulation of differentiation and lineage specification has been substantiated *in vitro* in endometrial carcinoma cell lines. In summary, this research identified *GPC5* amplification as the molecular event mediating the epithelial-mesenchymal transition (EMT) in a subset of endometrial carcinosarcomas (Wang et al., 2016; Veugelers et al., 1997). *GPC5* plays an essential role in fundamental cellular processes, including normal cell division, growth, proliferation, and development (Yang and Wang, 2023; Mukherjee et al., 2023).

Furthermore, *GPC5* is pivotal in orchestrating the initiation, proliferation, migration, and invasion of cancer cells by activating signaling pathways such as Wingless/Integrated (Wnt), aryl hydrocarbon receptor (AHR) and Hedgehog (Hong et al., 2019; Yang et al., 2021; Li et al., 2011). Disturbances in both the genomic locus and expression levels of *GPC5* have been reported across a spectrum of human cancers, such as breast cancer (Kiely et al., 2021), lymphoma (Yu et al., 2003), rhabdomyosarcoma (Chui et al., 2018), glioma (Müller et al., 2023), prostate cancer (de Moraes et al., 2021) and pancreatic cancer (Liu et al., 2020).

Thus, investigating *GPC5* mutations that may be connected to the development of lung adenocarcinoma is vital, offering novel insights into its role in the disease’s pathogenesis. In this study, we identified a rare germline mutation in *GPC5* (c.776C>T) in a LUAD pedigree and examined its effect on the tumor-suppressing functions of *GPC5*.

## Materials and methods

### Patient recruitment and exome sequencing

The Ethical Committee of Peking Union Medical College Hospital approved this study, the data from this study have been uploaded to the SRA database (BioProject ID: PRJNA1240214) and will be made publicly available, solely for scientific research within the applicable scope. All participants provided written informed consent, ensuring that the utilization of biological materials and study procedures adhered to Helsinki Declaration guidelines and regulations. DNA from germline was extracted using Biomed DNA Blood Mini Kit (Biomed, Japan) from patients’ peripheral blood with. DNA extraction from formalin-fixed, paraffin-embedded (FFPE) tissues was performed using Qiagen GeneRead kits (Qiagen, United States). Whole-exome sequencing (WES) libraries were prepared with SureSelect Human All Exon V7 kit (Agilent Technologies, United States). Sequencing was conducted on the NovaSeq 6,000 platform (Illumina, United States) using paired-end mode (2 × 150 bp), achieving a final on-target coverage of 100×. WES data processing was performed following previously established methods (Zhao et al., 2021; Wang et al., 2022; Chen et al., 2021). For mutation nomenclature, we referred to the RefSeq accession number *NM_004466.6* for the *GPC5* transcript and corresponding protein isoform.

### Construction and transfection of plasmid

The cDNA of the human *GPC5* gene (NM_004466.6) was subcloned into the pEGFP-N1 vector (Clontech, United States) to create the recombinant vector pEGFP-N1-*GPC5*. This vector was used as the template for site-directed mutagenesis using KOD-NEO-PLUS Kit (Takara, Japan), according to the manufacturer’s instructions. The primers used for site-directed mutagenesis are detailed in Supplementary Table 1. Both strands of the mutant plasmids were sequenced to confirm the nucleotide mutation.

### Cell lines and cell culture

A549 and H2009 cells were kindly provided by Institute of Basic Medical Sciences at the Chinese Academy of the Medical Sciences (Catalog Number: 1101HUM-PUMC000002, 1101HUM-PUMC000002). Prior to experiment, *mycoplasma* detection was conducted, yielding negative results. A549 cells were cultured in McCoy’s 5A medium (Gibco, United States) with 10% fetal bovine serum (FBS) (HyClone, United States), 100 μg/mL streptomycin, and 100 U/mL penicillin in 5% CO_2_ atmosphere at 37°C.H2009 cells are cultured in DMEM/F12 medium supplemented with 5% fetal bovine serum, 1% GlutaMAX-1, 1% penicillin-streptomycin, 1% ITS (insulin-transferrin-selenium), 10 nM hydrocortisone, and 10 nM β-estradiol, and maintained in an environment of 95% air and 5% carbon dioxide. When A549 and H2009 cells reached 65% confluency, they were transfected with 2 µg of pEGFP-N1-*GPC5*-WT or mutant plasmid using Lipofectamine 3,000 (Life Technologies, United States).

### Western blot analysis

48 h after transfection, A549 and H2009 cells transfected with WT or mutant *GPC5* plasmid were rinsed with cold phosphate-buffered saline (PBS) and lysed with 100 µL RIPA buffer (Sigma, United States) supplemented with protease inhibitor cocktail (Sigma, United States). 24 h prior, the secreted protein was substituted with serum-free medium. The cell supernatant was collected by centrifugation at 3,000 **×** g for 15 min, then concentrated using centrifugal filter (NMWL: 3K) (MERK, Germany) by centrifuging at 4000g for 30 min. The concentration process was repeated until the total volume reached approximately 400 µL. 30 μg total proteins were separated via SDS-PAGE, and transferred to PVDF membrane (Millipore, United States). Membranes were blocked with 10% skim milk at 24°C for 3 h, followed by an incubation with primary antibodies for 12 h at 4°C. Details of the primary antibodies and antibody working concentration used can be found in Supplementary Table 2. Following three washes with TBST (Tris-buffered saline with Tween 20), membranes were incubated with secondary antibodies (Cell Signaling Technology, United States) at 24°C for 1 h. Proteins were visualized using ChemiDoc MP Imaging System (Bio-Rad, United States).

### RNA extraction and real-time PCR

Total RNA from A549 and H2009 cells transfected with WT or mutant *GPC5* plasmid was extracted using TRIzol reagent (Takara, Japan). Reverse transcription was performed using the QuantiNova Reverse Transcription Kit (Baocheng, China). Quantitative PCR was conducted on a CFX Opus96 system (Biorad, United States) using SYBR Green I Mix (HANCHBIO, China). The relative mRNA relative expression levels were calculated using the cycle threshold (Ct) method, normalized to the level of GAPDH, and analyzed using the 2^−ΔΔCT^ formula. Primer sequences were provided in Supplementary Table 3.

#### Cell viability assay and colony formation assay

The Cell counting kit-8 (CCK-8) assay was performed as previously described (Niu et al., 2018). A549 and H2009 cells transfected with WT or mutant *GPC5* plasmids were seeded in 96-well plates at a density of 3,000 cells per well and cultured for 5 days. Each day, 10 μL of CCK-8 reagent (KeyGEN, China) was added to each well and incubated for 2 h. Cell viability was evaluated by measuring the optical density at 450 nm. Then, for the colony formation assay, transfected A549 cells were cultured in 6-well plates at a density of 2000 cells per well for 14 days. After fixation with methanol and staining with 0.25% crystal violet, colonies were counted were counted. All experiments were performed in triplicate, and the data are expressed as mean ± SEM.

#### EdU assay

Cell proliferation was assessed using the EdU Cell Proliferation Kit. A549 cells transfected with *GPC5*-WT, *GPC5*-Mutant or *GPC5*-siRNA plasmids were plated at a density of 20,000 cells per well in 24-well plates containing pre-embedded cell slides and cultured for 3 days. Cells were treated using BeyoClick™ EdU Cell Proliferation Kit with Alexa Fluor 594 (Beyotime Biotechnology, China). Proliferation was quantified using Laser Scanning Confocal Microscopy (Nikon A1, Japan). All experiments were conducted in triplicate, and the data are expressed as mean ± SEM.

#### TUNEL assay

One-step TUNEL apoptosis assay kit was applied to detect the cell proportion of cell apoptosis. For cell apoptosis assay, A549 cells transfected with *GPC5*-WT, *GPC5*-Mutant or *GPC5*-siRNA plasmids were plated at a density of 20,000 cells per well in 24-well plates pre-embed cells into a cell slide and cultured for 3 days. Cells were treated with the BeyoClick™ One Step TUNEL Apoptosis Assay Kit containing Alexa Fluor 594 (Beyotime Biotechnology, China). Apoptotic activity was evaluated using Laser Scanning Confocal Microscopy (Nikon A1, Japan). Experiments were repeated three times, and the data are reported as the mean ± SEM.

#### Wound healing assay

A549 and H2009 cells transfected with either wild-type or mutant *GPC5* plasmid were plated in a 6-well plate at 2 × 10^6^ cells per well and grown to confluence. The cells were then treated with 0.2 mg/mL Mitomycin C (MERK, Germany) for 10 min. A linear scratch was made with a pipette tip to mimic a wound. The culture medium was replaced by McCoy’s 5A medium, and Wound closure was monitored at 0 and 12 h post-scratch using a ×10 magnification.

#### Cell migration and invasion assay

7 × 10^4^ A549 and H2009 cells transfected with WT or mutant *GPC5* plasmid were cultured in the upper chamber in 400 μL McCoy’s 5A medium, and 600 μL of McCoy’s 5A medium with 10% FBS was added to the lower chamber. After 24–48 h, a wet cotton swab was used to remove the cells that remained in the upper chamber. The chambers were then fixed with methanol and stained with 0.2% crystal violet. Invading cell number was counted in five randomly chosen microscopic fields. Approximately 2 × 10^5^ A549 cells were utilized for the cellular invasion assay. Cell invasions were assessed by Transwell chambers coated with Matrigel (Corning, United States), following a previously published method.

#### Flow cytometry

A549 cells were seeded in 6-well plates (2 × 10^6^ cells per well) and transfected with WT or mutant *GPC5* plasmid. Prior to flow cytometry analysis, cells were incubated in serum-free medium for 24 h h. For apoptosis analysis, A549 cells were stained with propidium iodide (PI) and Annexin V using an apoptosis detection kit (4A Biotech, China). Cells were fixed in cold 70% ethanol at 4°C for 4 h followed by PI staining (Sigma, United States) for cell cycle analysis.

### Immunohistochemical staining

Formalin-fixed, paraffin-embedd (FFPE) tissues and H&E-stained sections were provided by the pathology department and reviewed by a consultant histopathologist. For immunohistochemical (IHC) staining, FFPE tissue sections underwent deparaffinization through a graded ethanol series, followed by rehydration. The sections were then treated with methanol containing 0.3% hydrogen peroxide for 10 min at room temperature to block endogenous peroxidase activity. Antigen retrieval was performed by heating the slides in a pH 6.0 retrieval buffer for 30 min. Slides were incubated overnight at 4°C with either an anti-*GPC5* antibody or control immunoglobulin G (IgG; 1 mg/mL). After washing with PBS, detection was carried out using Prolink-2 Plus HRP rabbit polymer detection kit (Merck, Germany). Images were scanned with Leica Aperio AT2 scanner (Leica, Germany).

### Statistical analysis

Western blot images were analyzed using ImageJ (NIH, United States) was used to analyze Western blotting images for quantifying the relative optical density of proteins in this study, reflecting expression levels. Overall expression levels of WT and mutant *GPC5* were normalized to GAPDH levels. Each experiment was repeated three times. For the imaging results, a representative image from representative images from one experiment are shown. The statistical analysis was conducted with GraphPad Prism 9.0 software (GraphPad Software, United States). The Mann-Whitney U test was applied to evaluate the differences between two groups, while ANOVA with *post hoc* tests was employed to compare differences among multiple groups through *post hoc* tests. Significance thresholds: *P < 0.05, **P < 0.01, ***P < 0.001, and ****P < 0.0001.

## Results

### Elucidating *GPC5* mutations in the LUAD pedigree

**FIGURE 1:**
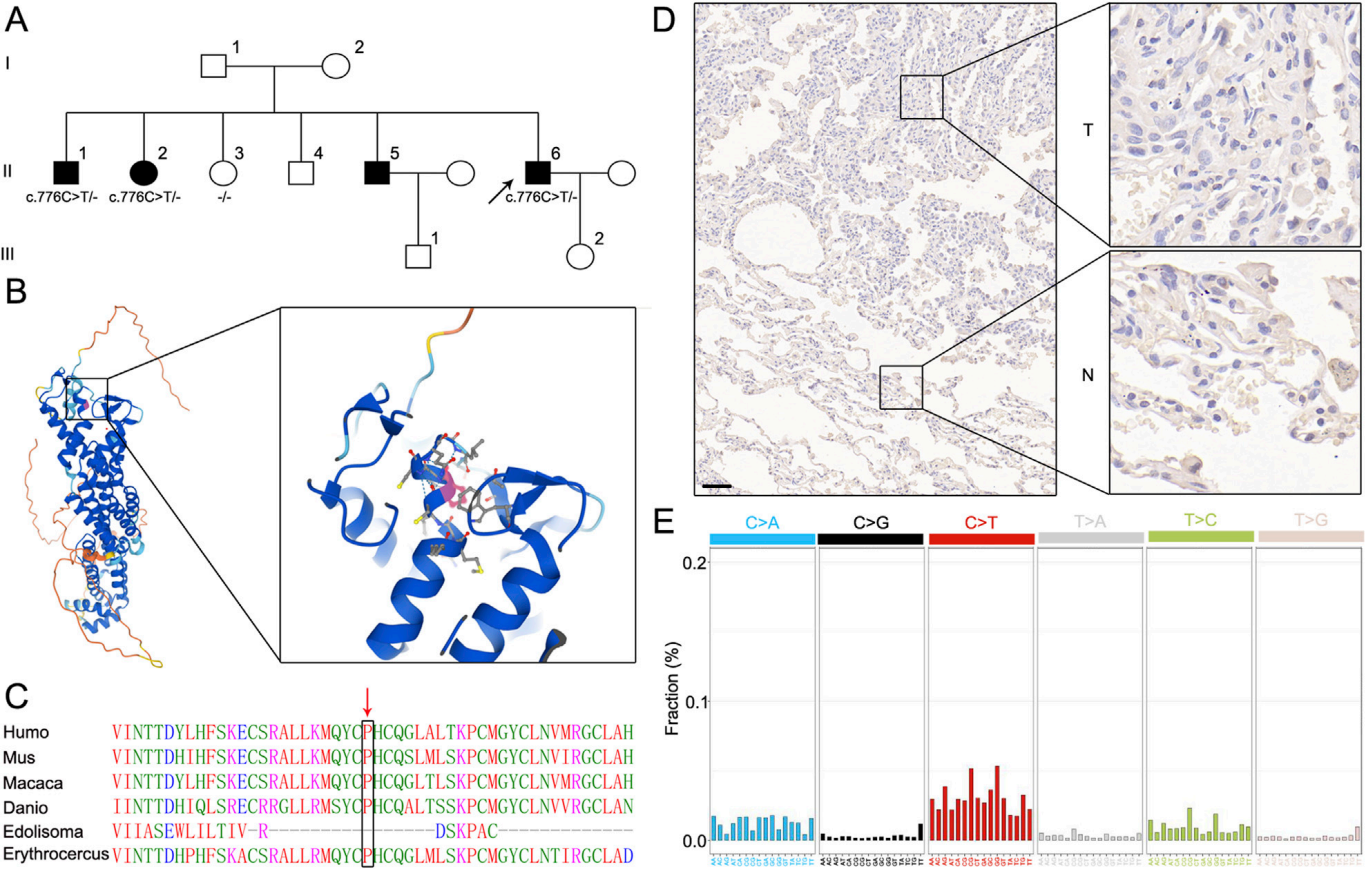
A newly identified lung adenocarcinoma-related *GPC5* germline mutation. **(A)** Pedigree of a family with multiple cases of lung adenocarcinoma. **(B)** Spatial distribution of the variant in a 3D model using AlphaFold Protein Structure Database (https://alphafold.ebi.ac.uk/). **(C)** Position of the variant relative to an evolutionarily conserved region using ClustalW sequence alignment (https://www.genome.jp/tools-bin/clustalw). **(D)** Immunohistochemical staining of *GPC5* protein expression in proband LUAD tissues (T) and adjacent normal tissues (N) from II 6; Scale bar = 100 μm. **(E)** Patterns of mutation signature observed in proband genomes using the Wellcome Trust Sanger Institute mutational signatures framework.

We recruited a Chinese family with four members affected by LUAD (Figure 1A). The proband (II-6) was diagnosed with LUAD at 53 years of age with a 0.5 cm *in situ* adenocarcinoma in the left lung. Two older brothers (II-1 and II-5) and one sister (II-2) of the proband were diagnosed with LUAD at ages 58, 60, and 66, respectively. All affected members were nonsmokers. Exome sequencing (ES) was performed on peripheral blood DNA from 3 affected members (II-1, II-2, and II-6) and one healthy member (II-3, age 46) in the pedigree. First, we analyzed rare (minor allele frequency <1%) variants carried by all three affected members. To that end, we manually curated variants in disease databases, such as The Catalogue of Somatic Mutations in Cancer (COSMIC) and Human Gene Mutation Database (HGMD), and determined whether the mutated genes had biological relevance to oncogenesis. As a result, we prioritized *GPC5 c.776C>T* (*p.Pro259Leu*) in *GPC5*, a known tumor suppressor gene for lung cancer. The resulting protein change alters an evolutionarily conserved sequence (Figures 1B,C), and was predicted as pathogenic by Polyphen-2 (Hum Div score = 1). This missense variant was not carried by the healthy family member (II-3) and is extremely rare in the Genome Aggregation Database (1/250,688, max population frequency = 0.000003989). In the FFPE tumor sample of patient II-6, immunohistochemistry revealed *GPC5* expression showed a slightly reduced expression level of *GPC5* in the tumor sample versus normal lung (Figure 1D), consistent with previous studies. In addition, we performed ES on the FFPE tumor and identified no “second-hit” *GPC5* variants. Despite a dominant C>T mutational pattern, we did not identify a significant somatic mutational signature in the tumor tissue (Figure 1E).

### *In vitro* evaluation of the cellular biological consequences of *GPC5* mutation

**FIGURE 2:**
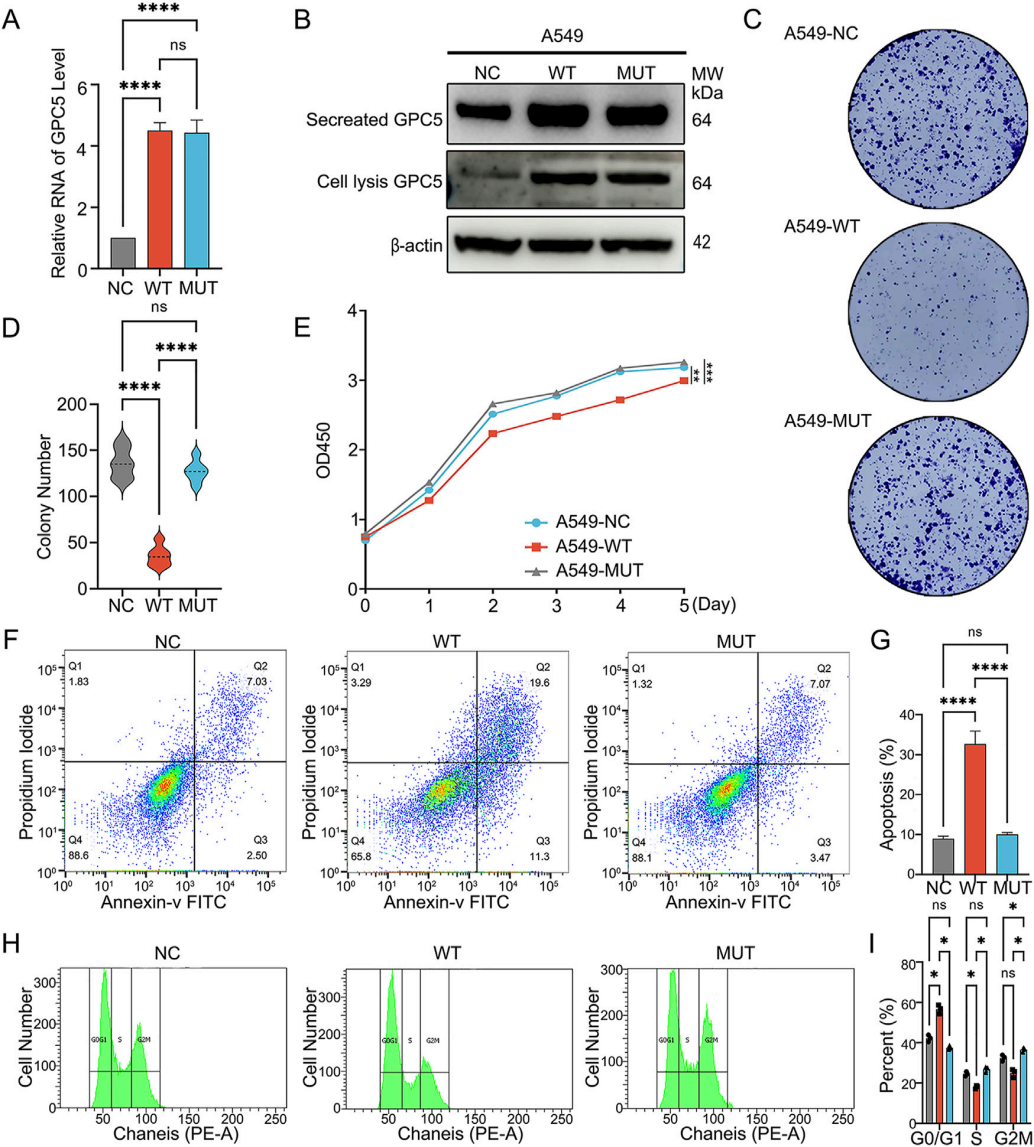
The *GPC5* germline mutation. (c.776C>T) reverses its tumor-suppressing functions. **(A,B)** *GPC5* mRNA and protein levels in A549-*NC*, A549-*WT*, A549-*MUT* groups were assessed by qPCR and Western blotting. *GPC5* mRNA levels were normalized to GAPDH, while protein levels were normalized to β-actin. **(C**,**D)** Assessment of cell clonogenicity in A549-*NC*, A549-*WT*, A549-*MUT* groups were measured by colony formation assays **(E)** Evaluation of proliferative capacity in A549-*NC*, A549-*WT*, A549-*MUT* groups via CCK-8 assays. **(F,G)** Apoptosis analysis in A549-NC, A549-WT, and A549-MUT groups was performed using Annexin V and PI staining **(H,I)** Flow cytometric cell cycle analyses were conducted using A549-NC, A549-WT, and A549-MUT groups. NC: Negative control. Data are shown as the mean ± SEM of three independent experiments. *P < 0.05, **P < 0.01, ***P < 0.001 and ****P < 0.0001 by two-way ANOVA.

In order to choose a non-small cell lung cancer cell line for subsequent experiments, we employed various non-small cell lung cancer cell lines to measure the protein expression level of *GPC5* via Western blot assays. The outcomes indicated that the A549 and H2009 cell lines exhibited the lowest *GPC5* expression levels (Supplementary Figure 1). To ascertain the impact of variants on the tumor-suppressive functions of *GPC5*, to assess the variant’s impact by transfecting LUAD A549 cell lines with vectors expressing *GPC5* wild-type (WT) or the c.776C>T mutant (MUT). As demonstrated by qPCR and Western blot analyses, qPCR and Western blot confirmed elevated in *GPC5* mRNA and protein levels within MUT *GPC5* and WT *GPC5* groups compared to the NC group; however, mRNA (Figure 2A) and protein expression levels (Figure 2B) were comparable between MUT *GPC5* and WT *GPC5* groups. To investigate the influence of wild-type (WT) *GPC5* or the c.776C>T mutant (MUT) on the proliferation capacity of A549 cells, a colony formation assay was conducted. The results indicated a notably reduced number of colonies in *GPC5* WT group compared to NC group, whereas *GPC5* MUT group exhibited a significantly increased number of colonies, but MUT significantly increased relative to *GPC5* WT group (Figures 2C,D). Additionally, CCK-8 assays revealed reduced proliferation in *GPC5* WT group, while the *GPC5* MUT group demonstrated a significant rise in proliferative capacity compared to *GPC5* WT group (Figure 2E),suggesting the mutation attenuates c.776C>T mutation in *GPC5* substantially attenuates its inhibitory effect on the proliferation of A549 cells.

Flow cytometry for apoptosis of *GPC5* wild-type (WT) or c.776C>T mutant (MUT) on apoptosis in A549 cells, flow cytometry assays were conducted. Compared to the NC group, both early and late apoptosis were markedly increased in *GPC5* WT group, whereas in *GPC5* MUT group, MUT reduced apoptosis compared to *GPC5* WT group (Figures 2F,G), indicating that c.776C>T mutation in *GPC5* significantly diminishes its pro-apoptotic effect on A549 cells.

To examine the effects of *GPC5* wild-type (WT) or c.776C>T mutant (MUT) on the cell cycle in A549 cells, flow cytometry was employed. Relative to NC group, there was a significant increase in G1/S phase arrest in *GPC5* WT group, while a marked reduction was observed in *GPC5* MUT group compared to *GPC5* WT group (Figures 2H,I), indicating that c.776C>T mutation in *GPC5* drastically reduces its capacity to induce cell cycle arrest in A549 cells.

**FIGURE 3:**
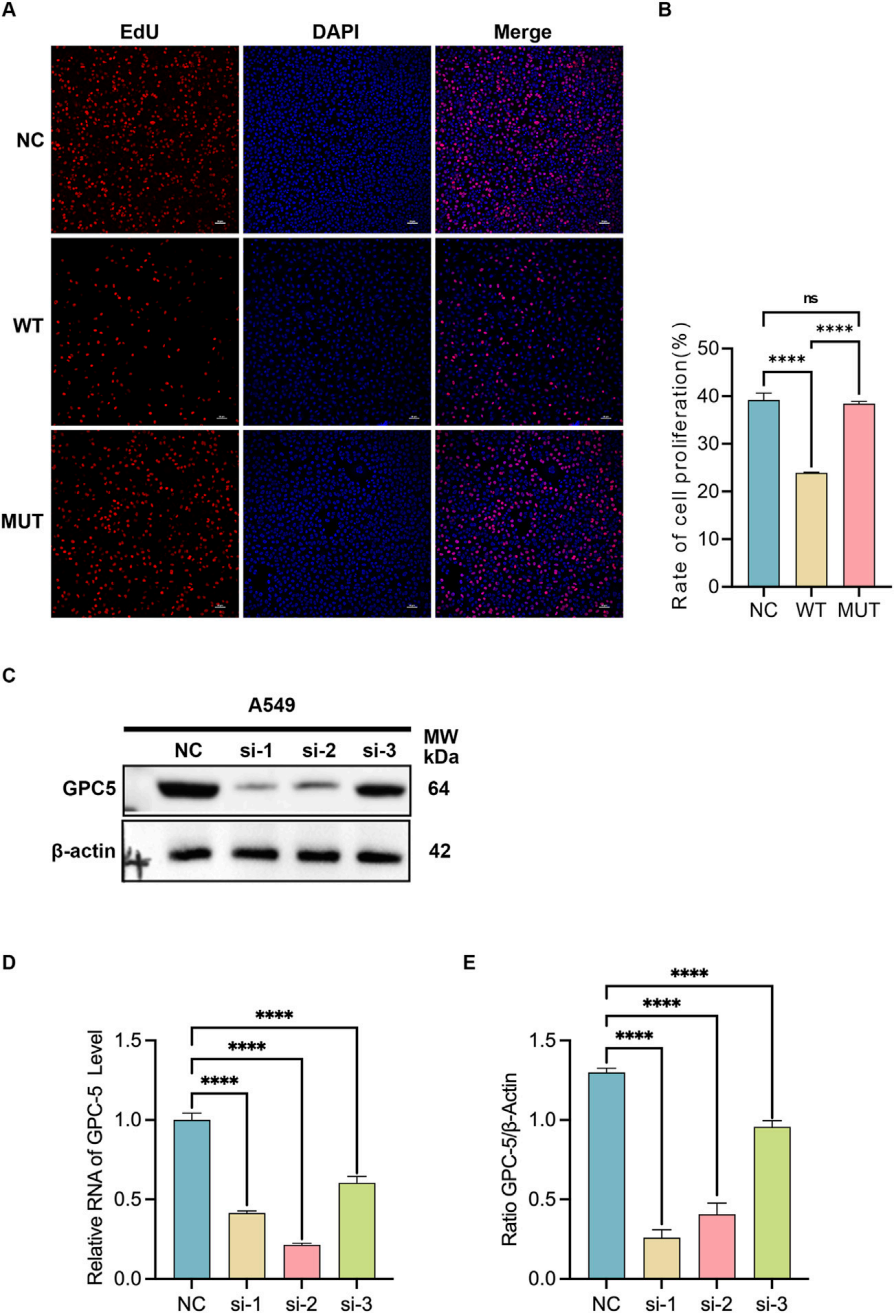
*GPC5* mutation enhances the proliferative capacity of A549 cells and verifies the efficacy of *GPC5*-si silencing. **(A,B)** Graphical representation and statistical analysis of cell proliferation ratios in A549 cells from NC, *GPC5*-WT, and *GPC5*-MUT groups were determined by EdU assay. NC: Negative control. **(C,E)** Graphical representation and statistical analysis of *GPC5* protein expression levels in A549 cells from NC, *GPC5*-si-1, *GPC5*-si-2, and *GPC5*-si-3 groups were assessed by Western blot analysis. NC: Negative control. **(D)** Graphical representation and statistical analysis of *GPC5* mRNA expression levels in A549 cells from NC, *GPC5*-si-1, *GPC5*-si-2, and *GPC5*-si-3 groups were determined by RT-PCR. NC: Negative control. Data are presented as the mean ± SEM of three independent experiments. *P < 0.05, **P < 0.01, ***P < 0.001 and ****P < 0.0001, as analyzed by one-way ANOVA.

To further validate the influence of *GPC5* wild-type (WT) or c.776C>T mutant (MUT) on proliferative abilities in A549 cells, an EdU cell proliferation assay was conducted. The results showed a significant decrease in EdU-positive cells in *GPC5* WT group compared to NC group. However, *GPC5* MUT group showed a marked increase compared to *GPC5* WT group (Figures 3A,B). EdU To delve deeper into the role of *GPC5* in A549 cells, ***GPC5-siRNAs (si-1, si-2)*** were synthesized and transfected into A549 cells. Western blot and RT-qPCR analyses validated the knockdown efficiency of *GPC5*-siRNAs, confirmed knockdown efficiency (Figures 3C,D, E).

**FIGURE 4:**
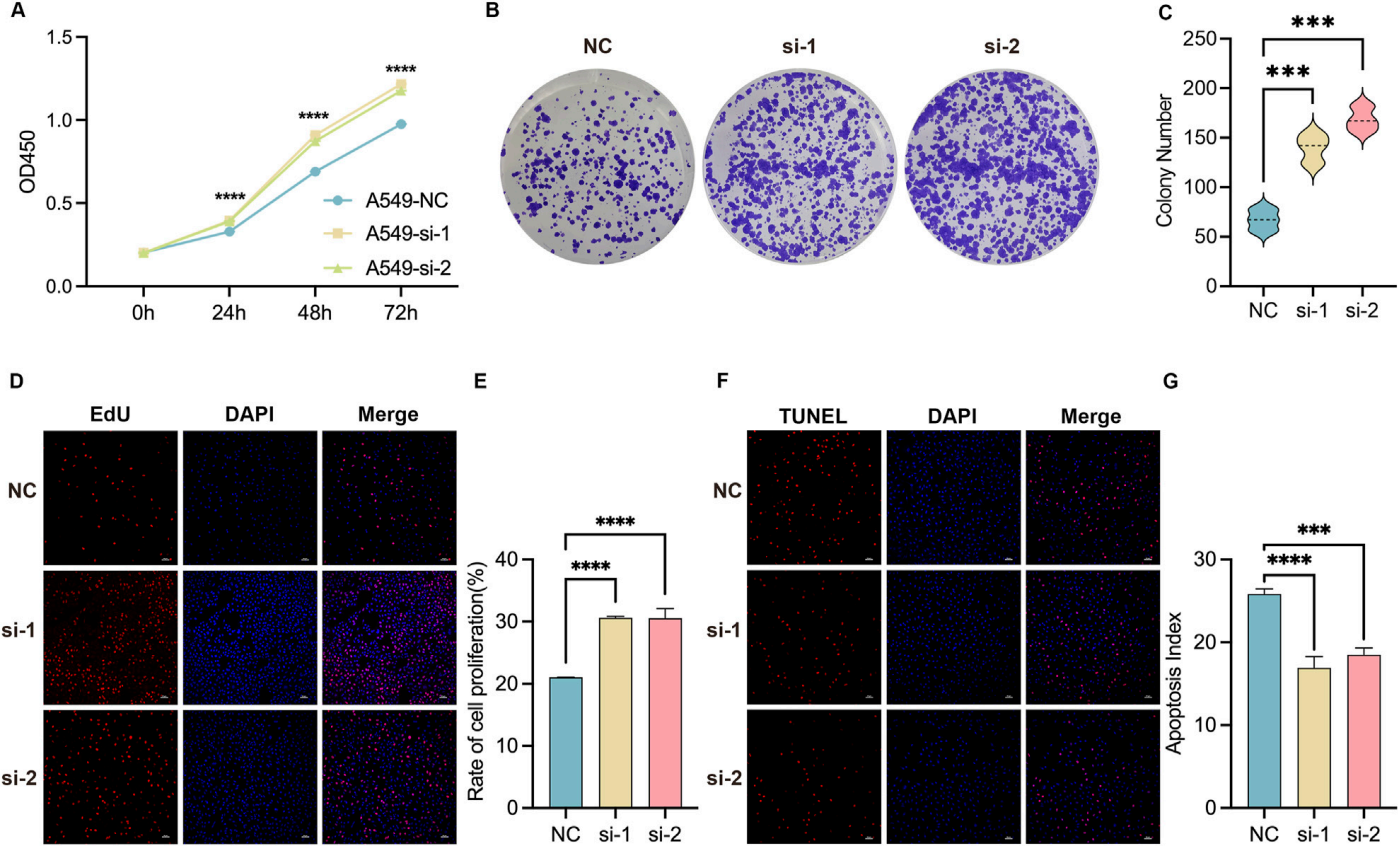
The silencing of *GPC5* promotes the proliferative capacity of A549 cells and impedes their apoptotic potential. **(A)** Assessment of cell proliferation capabilities in NC, *GPC5*-si-1, and *GPC5*-si-2 groups *via* CCK8 assay. **(B,C)** Graphical representation and statistical analysis of colony formation by A549 cells in NC, *GPC5*-si-1, and *GPC5*-si-2 groups, as evaluated by colony formation assay. **(D,E)** Graphical representation and statistical analysis of proliferation ratios in A549 cells from NC, *GPC5*-si-1, and *GPC5*-si-2 groups, as detected by EdU assay. **(F,G)** Graphical representation and statistical analysis of apoptosis ratios in A549 cells from the NC, *GPC5*-si-1, and *GPC5*-si-2 groups, as measured by TUNEL assay. Data are presented as the mean ± SEM of three independent experiments. NC: Negative control. *P < 0.05, **P < 0.01, ***P < 0.001 and ****P < 0.0001, as determined by two-way ANOVA.

CCK-8 assays was demonstrated that compared to si-NC group, increased viability in *GPC5*-si-1 and *GPC5*-si-2 groups was significantly increased (Figure 4A). Subsequent experiments using *GPC5*-si-1 and *GPC5*-si-2 showed that number of single-cell colonies increased significantly compared to si-NC group (Figures 4B,C). EdU assays also indicated an increase in EdU-positive cells compared to si-NC group (Figures 4D,E), demonstrating that *GPC5* depletion augments the proliferative capability of A549 cells. TUNEL immunofluorescence assays revealed a reduction in apoptotic cells compared to si-NC group (Figures 4F,G), indicating that *GPC5* knockdown reduces apoptosis levels in A549 cells.

### *In vitro* evaluation of the influence of mutated *GPC5* on migration and invasion capacities

**FIGURE 5:**
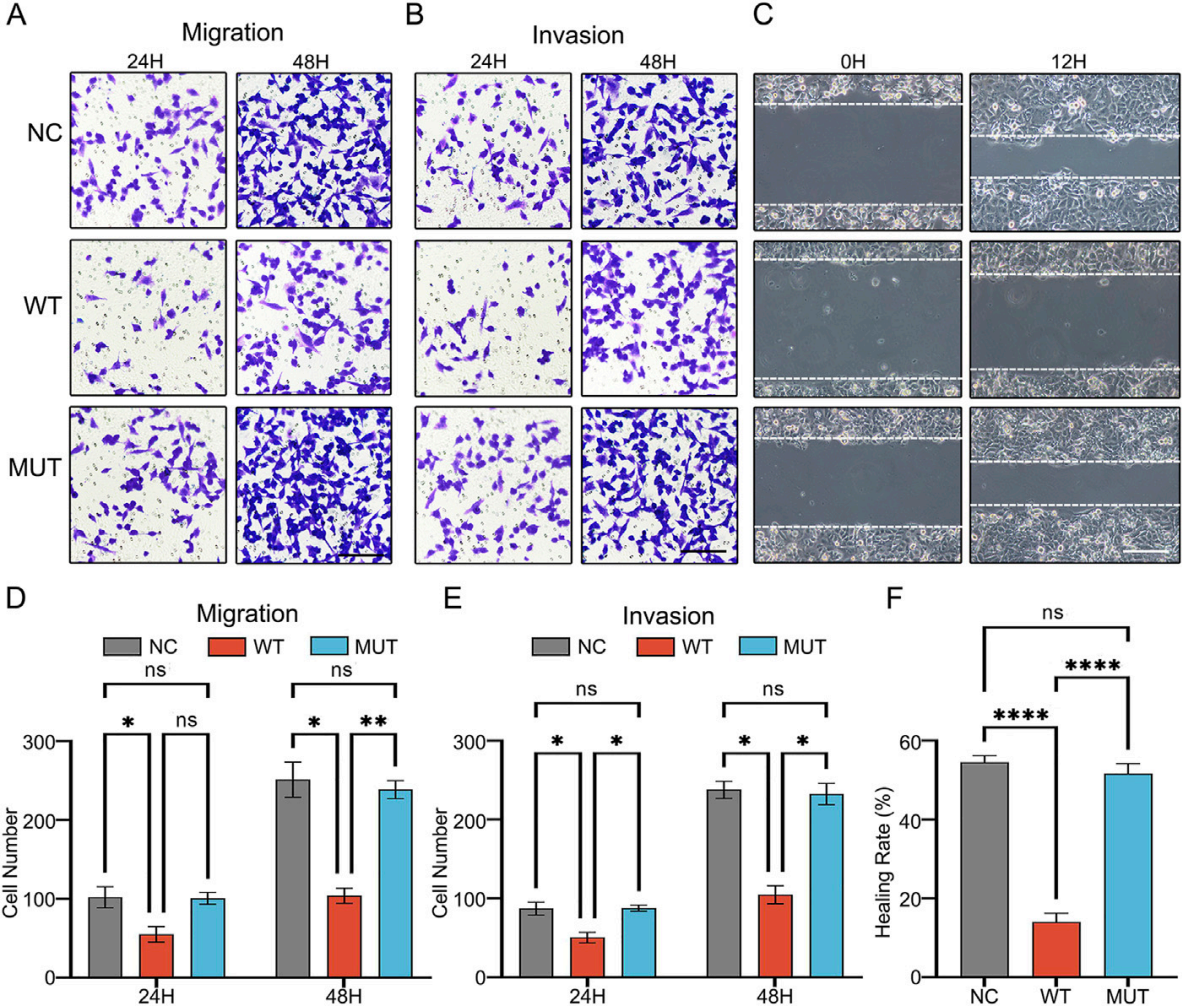
The novel *GPC5* germline mutation (c.776C>T) promoted tumor migration and invasion. **(A,B,D,E)** Cell migration and invasion were tested by Transwell and **(C,F)** wound-healing assays in A549-*NC*, A549-*WT*, A549-*MUT* groups. Scale bar = 100 μm. Data are shown as the mean ± SEM of three independent experiments. NC: Negative control. *P < 0.05, **P < 0.01, ***P < 0.001 and ****P < 0.0001 by two-way ANOVA.

Previous reports revealed that *GPC5* might modulate EMT by preventing β-catenin nuclear localization, thereby preventing lung cancer cells migration and invasion (Wang et al., 2016). To investigate the effects of wild-type (WT) *GPC5* or c.776C>T mutant (MUT) on the migratory and invasive capacities of A549 cells, we conducted Transwell assays with and without Matrigel. Compared to the NC group, the number of migrating and invading cells in the *GPC5* WT group was significantly reduced. However, in comparison to the *GPC5* WT group, the *GPC5* MUT group exhibited a marked increase in cell migration (Figures 5A,D) and invasion (Figures 5B,E), suggesting that the c.776C>T mutation in *GPC5* significantly enhances its ability to promote the migration and invasion of A549 cells. To further validate these findings, we performed a wound-healing assay, which showed a markedly shorter migration distance in *GPC5* WT group compared to the NC group, but an increased migration distance in *GPC5* MUT group relative to *GPC5* WT group (Figures 5C,F). The same experiment was validated in another cell line, H2009 (Supplementary Figure 2).

**FIGURE 6:**
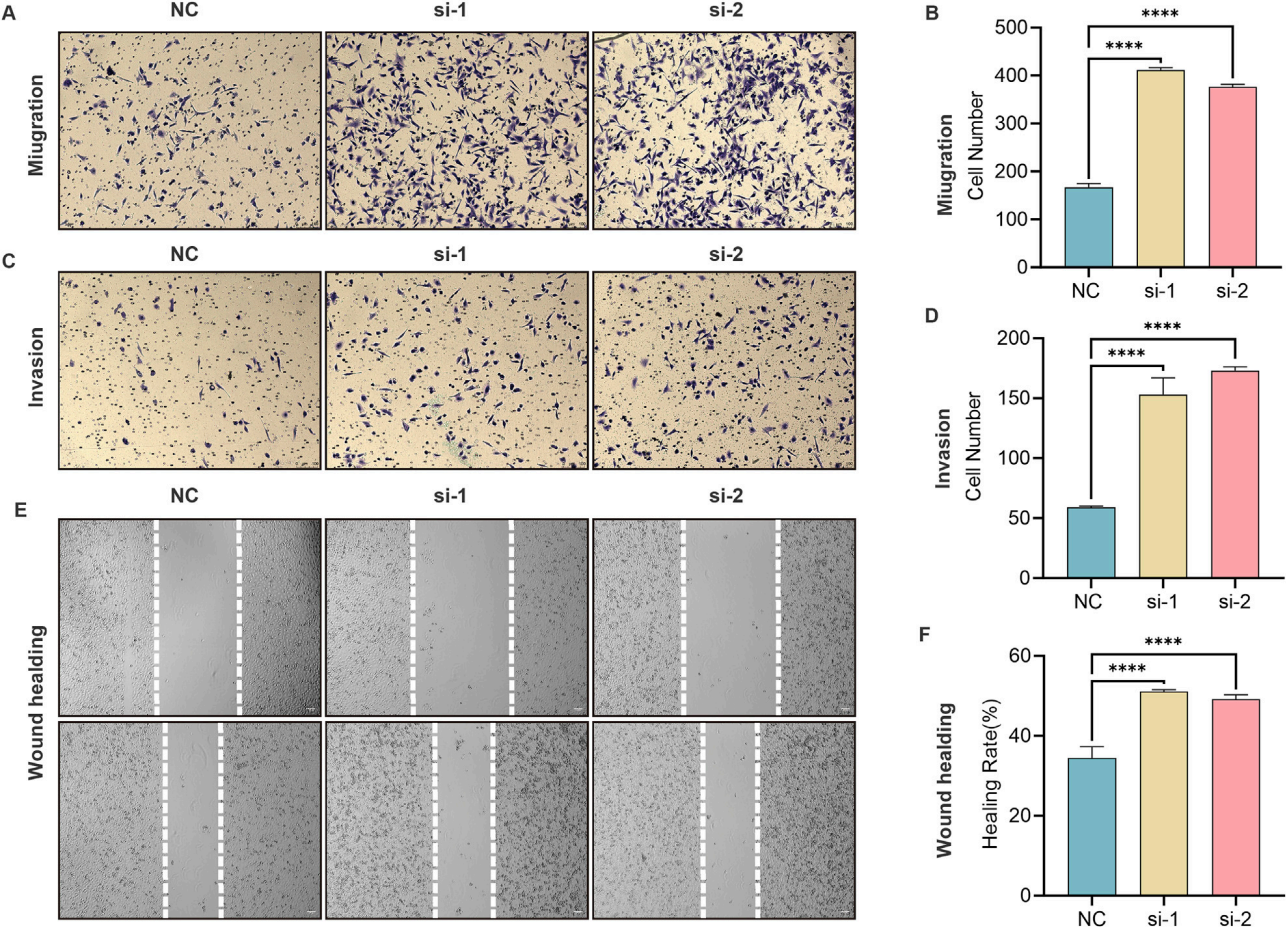
Silencing of *GPC5* augments the migratory and invasive capabilities of A549 cells. **(A,B)** Graphical representation and statistical analysis of the number of migratory A549 cells in NC, *GPC5*-si-1, *GPC5*-si-2 groups, as evaluated by Transwell assay. **(C,D)** Graphical representation and statistical analysis of the number of invasive A549 cells in NC, *GPC5*-si-1, *GPC5*-si-2 groups, as assessed by Transwell assay. **(E,F)** Graphical representation and statistical analysis of migration distances of A549 cells in NC, *GPC5*-si-1, *GPC5*-si-2 groups, as measured by scratch assay. Data are presented as the mean ± SEM of three independent experiments. NC: Negative control. *P < 0.05, **P < 0.01, ***P < 0.001 and ****P < 0.0001, as analyzed by two-way ANOVA.

To substantiate the migratory and invasive capacity of *GPC5*-knockdown A549 cells, Transwell experiments revealed that both *GPC5*-si-1 and *GPC5*-si-2 groups demonstrated a significant increase in migrating and invading cells compared to si-NC group (Figures 6A–D). The wound-healing assay also exhibited a noticeable increase in migration distance in *GPC5*-si-1 and *GPC5*-si-2 groups compared to si-NC group (Figures 6E,F).

### *In vitro* assessment of mutant *GPC5* effects on EMT and Wnt signaling pathway

**FIGURE 7:**
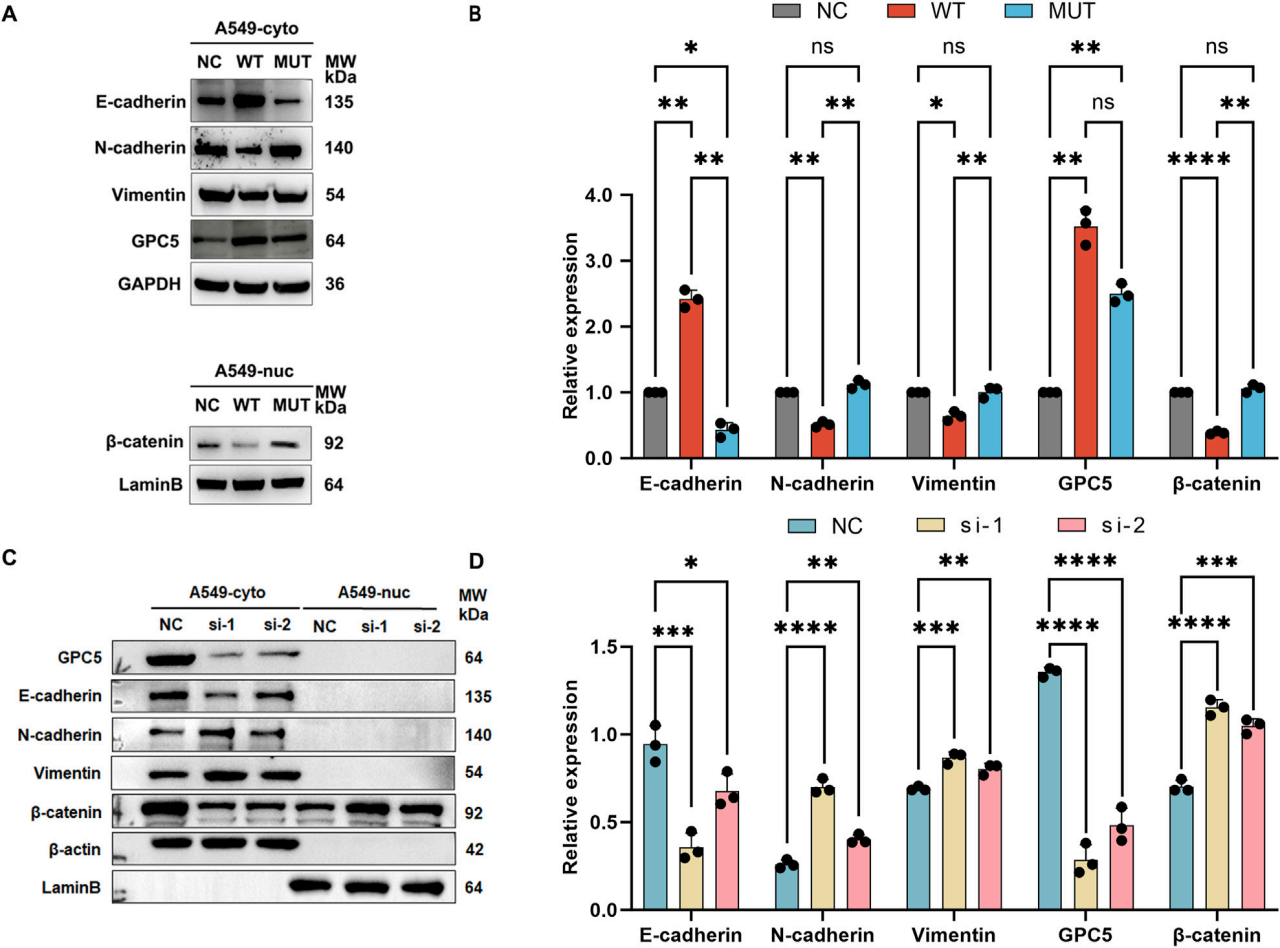
The novel *GPC5* germline mutation (c.776C>T) and *GPC5* silencing promoted epithelial-to-mesenchymal transition. **(A,B)** Western blot analysis of EMT markers (E-Cadherin, N-Cadherin, Vimentin) and β-catenin in A549-*NC*, A549-*WT*, A549-*MUT* groups. Protein levels were normalized to GAPDH, and nuclear β-catenin levels were normalized to lamina B. **(C**,**D)** Western blot analysis of *GPC5*, EMT markers, and β-catenin in A549 cells from NC, *GPC5*-si-1, and *GPC5*-si-2 groups. NC: Negative control. Data are presented as the mean ± SEM of three independent experiments. *P < 0.05, **P < 0.01, ***P < 0.001 and ****P < 0.0001, as determined by two-way ANOVA.

Consistently, protein level alteration of EMT-related genes displayed a consistent trend, in an *in vitro* assessment of mutant *GPC5*’s impact on EMT and the Wnt signaling pathway in A549 cells, protein level alterations of EMT-related genes exhibited a consistent trend. By isolating cytosolic and nuclear proteins and performing Western blot analyses, it was found that in the extracted cytosolic proteins, *GPC5* and E-cadherin protein levels were significantly elevated in *GPC5* WT group compared to NC group, while N-cadherin and Vimentin protein levels were markedly reduced. In contrast, *GPC5* MUT group showed a significant decrease in *GPC5* and E-cadherin levels and an increase in N-cadherin and Vimentin levels compared to *GPC5* WT group. Regarding nuclear-extracted proteins, the β-catenin level in the nucleus of *GPC5* WT group was significantly lower compared to the NC group, yet more elevated in *GPC5* MUT group compared to *GPC5* WT group (Figures 7A,B), indicating that c.776C>T mutation in *GPC5* facilitates nuclear translocation of β-catenin, thus initiating EMT process. To further explore the role of *GPC5*, after silencing it, cytosolic and nuclear proteins were separated and extracted for Western blot analysis, revealing a notable decrease in *GPC5* and E-cadherin protein levels, with an increase in N-cadherin and Vimentin levels in *GPC5*-si-1 and *GPC5*-si-2 groups compared to si-NC group. In these groups, the cytosolic β-catenin protein was reduced, whereas the nuclear protein level was significantly elevated (Figures 7C, D), demonstrating that *GPC5* influences the retention of β-catenin within the cytoplasm. The same experiment we validated in another cell line, H2009 (Supplementary Figure 3).

**FIGURE 8:**
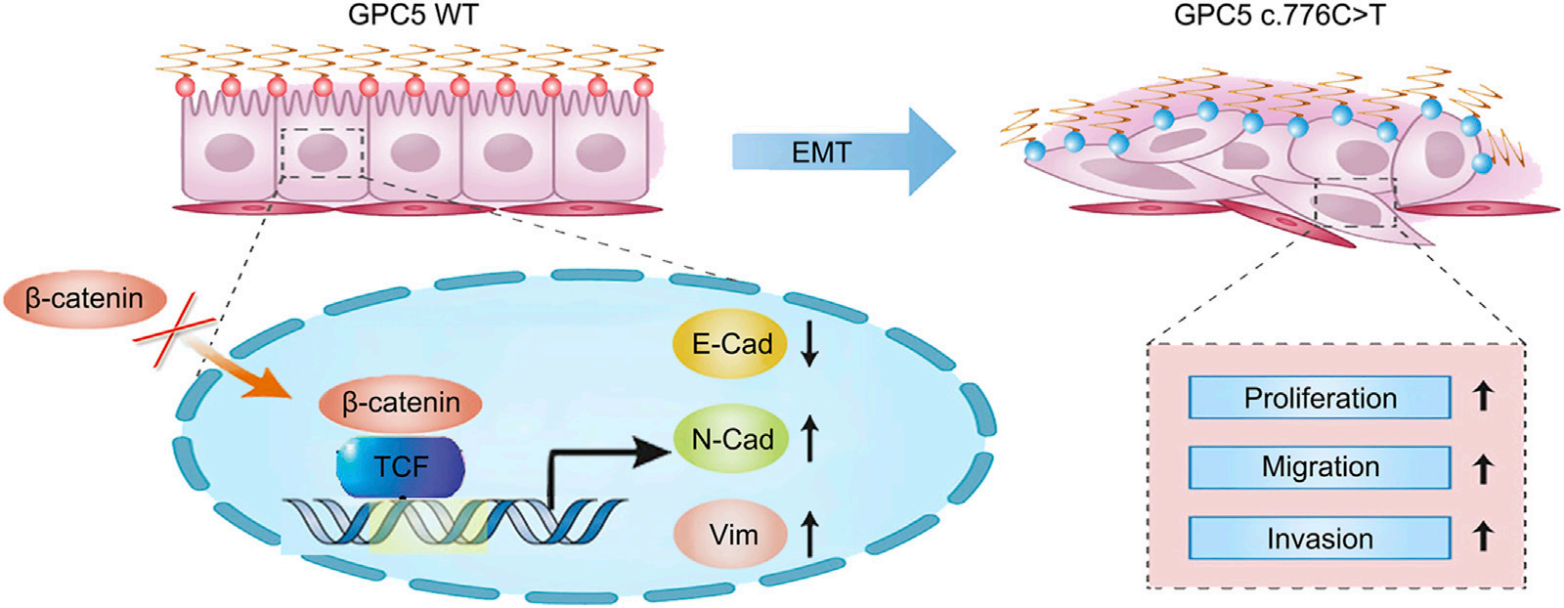
Schematic of the mechanism in which *GPC5* MUT promotes LUAD cell proliferation, migration, and invasion during tumor progression. *GPC5* MUT overexpression promotes nuclear localization of β-catenin, thereby downregulating E-cadherin (E-Cad) and upregulating N-cadherin (N-Cad) and vimentin (VIM). This induction of EMT, which leads to a loss of cell-cell adhesion and apical-basal polarity, accelerates cell proliferation, migration, and invasion. TCF: Transcription factor.

Our sequencing endeavors have unveiled the pivotal role of a single point mutation (c.776 C>T) within the *GPC5* gene. The c.776C>T mutation at *GPC5* locus inhibits the degradation of the protein complex responsible for β-catenin degradation, thereby resulting in an excess of β-catenin proteins translocating into the nucleus. Consequently, the nuclear translocation of β-catenin leads to an increase in nuclear β-catenin expression. Subsequently, nuclear β-catenin binds to transcription initiation sites, fostering the high expression of downstream proteins such as Vimentin and N-cadherin, which in turn promotes the proliferation, migration, and invasion of non-small cell lung cancer (Figure 8).

## Discussion

In essence, we focused on a Chinese family with multiple members affected by lung adenocarcinoma (LUAD), which provided a valuable opportunity to explore the genetic basis of familial LUAD. The fact that all the affected members were non-smokers is particularly notable, as it suggests that genetic factors may play a crucial role in the development of LUAD in this family, rather than the well-known environmental risk factor of smoking. We were the first to identify a germline mutation in *GPC5* (c.776C>T) among familial cases of LUAD, demonstrating that mutant *GPC5* undermines the tumor-suppressive functions of *GPC5*. Mutational sites of *GPC5* were identified through a bioinformatics database (Supplementary Figure 4). These findings suggest the potential genetic convergence of common and rare variations, underscoring *GPC5* as a plausible risk gene for LUAD.

Carcinogenesis is an intricate, multi-phase phenomenon influenced by numerous factors, with genetic mutations playing an integral role in cancer initiation and progression. While the majority of cancer mutation research emphasizes somatic mutations, germline mutations—located in reproductive cells and affecting all cells of the succeeding generation—have received less attention in the context of LUAD. To date, germline mutations linked to LUAD chiefly involve well-known oncogenes such as *BRCA2, EGFR, CHEK2, and ATR* (Eiamart et al., 2025; Bao et al., 2022; Wang et al., 2016).

Alterations in these genes are considered critical elements of FLC. In this study, we focused on germline mutations in genes related to LUAD and discovered a rare germline mutation in *GPC5* (c.776C>T) in three sibling LUAD patients. This *GPC5* missense variant has not been documented in genome or exome evaluations across combined populations. Subsequent analysis of *GPC5* expression in LUAD demonstrated a slight reduction in *GPC5* levels compared to normal tissue. The study has certain limitations. The single-family design restricts the sample size. This makes it possible that there is population-specific rarity in the samples we observed. Mutations in *GPC5* (c.776C>T) are not among the more common mutations in the clinical setting, making it difficult to collect additional samples. For this reason, it may be more appropriate for us to conduct follow-up in this family to determine whether the *GPC5* mutation has persisted over time, whether there will be other mutations, whether it is always present, or whether other mutations will appear.

Although we preliminarily verified that this rare *GPC5* germline mutation (c.776C>T) hastens LUAD progression, its physiological functions in tumor progression remain contentious. Prevailing studies have indicated that *GPC5* functions as a tumor suppressor gene, inhibiting cell proliferation and migration in lung cancer cells (Wang et al., 2016; Yang et al., 2021; Li et al., 2011; Yang et al., 2013).

To elucidate the mechanisms by which c.776C>T induces LUAD progression, we conducted *in vitro* experiments assessing the tumorigenic potential of this mutant protein. Our findings revealed that mutant *GPC5* did not compromise cell viability or anti-apoptotic capability as WT *GPC5* did, and the mutation did not disrupt cell cycle regulation. Consequently, we demonstrated that mutant *GPC5* attenuates the pro-apoptotic and cell cycle-arresting tumor suppressive effects of WT *GPC5*. Increasing evidence links EMT to cancer progression, metastasis, invasion, or drug resistance. Cancer cells undergoing EMT exhibit diminished expression of epithelial markers such as E-cadherin and elevated expression of mesenchymal markers like vimentin and N-cadherin. This transition enables the cells to reduce cell-cell adhesion and acquire enhanced invasive traits (Zhang et al., 2024).

Among the GPC family members, another highly homologous and extensively studied member, *GPC3*, has been proven to directly participate in modulating the Wnt signaling pathway (Capurro et al., 2005; Capurro et al., 2014) and Hedgehog signaling pathway (Capurro et al., 2008), making it a prime target in cell therapy (Saez-Ibañez et al., 2022). The Wnt/β-catenin signaling has been recognized as a critical player in the process of tumorigenesis (Zhi et al., 2025; Ge et al., 2025).

A previous study revealed that *GPC5* inhibited EMT via its mediation of Wnt/β-catenin signaling, likely due to its localization on the cellular membrane. This finding closely aligns with our results, as mutations in *GPC5* can bolster the nuclear translocation of β-catenin and elevate the expression of transcription factors integral to the EMT (Yang et al., 2021). Our sequencing efforts identified the pivotal role of a point mutation within the *GPC5* gene. Drawing upon both past literature and extant experimental findings, the c.776C>T mutation at this locus of *GPC5* is hypothesized to attenuate the intensity of its interaction with WNT3A, thereby enhancing the affinity of WNT3A for the LRP5/6 receptors. This augmented affinity promotes the strengthened binding of Dishevelled to GSK3β, PP2A, and APC, inhibiting the degradation of the protein complex responsible for β-catenin’s degradation. Consequently, β-catenin translocates into the nucleus, leading to an elevation in nuclear β-catenin expression. Nuclear β-catenin, in turn, binds to transcription initiation sites, enhancing the expression of downstream proteins such as Vimentin and N-cadherin. In summary, *GPC5*, a pivotal tumor suppressor gene across multiple malignancies, demonstrates a compromised anti-cancer efficacy post-mutation. Our examination confirmed c.776C>T reduced the *GPC5*-mediated prevention of β-catenin nuclear localization, which eventually induced changes in the expression of EMT markers and enhanced migration and invasion. We reorganized the alterations in β-catenin protein expression levels within both the nucleus and cytoplasm, thereby more prominently highlighting these changes in our result graphs. It has been observed that under different genetic contexts—such as *GPC5* WT versus MUT groups—the distribution of β-catenin between the nucleus and cytoplasm undergoes notable shifts, prompting an analysis of how these variations correlate with the EMT process and the activation of the Wnt signaling pathway. The data suggest that the c.776C>T mutation in *GPC5* expedites the translocation of β-catenin into the nucleus and initiates EMT, whereas the silencing of *GPC5* results in the accumulation of β-catenin within the nucleus, reducing its cytoplasmic retention in the cytoplasm. These analyses collectively underscore the regulatory role of *GPC5* in the localization of β-catenin and the morphological transformation of cells. We believe that it is highly necessary to conduct *in vivo* experiments. However, due to the limitations of the data obtained from *in vitro* experiments, the cellular mechanisms have not been fully explored. Therefore, we prefer to carry out *in vivo* experiments after conducting fairly comprehensive *in vitro* experiments. Considering that transgenic mice may have interference with *GPC5* due to their genetic background, if we can obtain lung cancer tissues carrying patient mutations again in the future, we will employ the patient-derived xenografts (PDX) model to further study the specific role and mechanism of the mutation in *in vivo* experiments.

Given the role of *GPC5* in regulating β-catenin signaling, patients carrying this germline mutation might benefit from combined therapies targeting both Wnt/β-catenin and immune checkpoints. For instance, inhibitors of β-catenin (PRI-724) or PD-1/PD-L1 antibodies (Zhou et al., 2023) could be explored in preclinical models of *GPC5*-mutant LUAD.

Given the pathogenic significance of GPC5 germline mutations in familial lung adenocarcinoma, we proposes a holistic management strategy for mutation carriers that integrates risk prevention, early detection, and precision therapy: genetic testing for GPC5 mutations should be prioritized in high-risk families, with genetic counseling to clarify risk stratification and establish long-term management plans for identified carriers, who are advised to undergo annual low-dose CT scans and sputum cytology tests starting at age 35 or 5–10 years before the earliest family-onset age, combined with dynamic monitoring of serum biomarkers like carcinoembryonic antigen, alongside primary prevention measures such as strict smoking cessation, occupational hazard protection, and lifestyle modifications to reduce cancer risk. This increased surveillance is essential given the potential role of the *GPC5* mutation in the development of LUAD. For carriers diagnosed with lung adenocarcinoma, comprehensive molecular profiling of tumor tissues—including somatic mutations (e.g., EGFR, KRAS), PD-L1 expression, and β-catenin status—is essential to exclude treatment resistance and guide targeted therapy, with patient-derived organoids or PDX models potentially utilized for pathway-specific drug sensitivity testing (e.g., Wnt inhibitors) and chemotherapeutic response evaluation when feasible; leveraging advancements in AI-driven imaging and biomarker analysis, as well as accelerated protein structure resolution, future research may expedite the discovery of effective therapeutics for GPC5-mutant tumors—such as allosteric inhibitors, antibody-drug conjugates, or chimeric antibody cell therapies—through molecular modeling and biological validation, enabling personalized treatment strategies tailored to the individual tumor biology of mutation carriers.

To date, this is the inaugural study confirming the c.776C>T germline mutation in *GPC5* as a genetic driver of familial LUAD. Importantly, our findings link *GPC5* dysfunction to Wnt/β-catenin activation and EMT, processes known to modulate immune evasion in LUAD. Thus, germline *GPC5* mutations may not only confer cancer risk but also predict immunotherapy resistance. Future studies should investigate whether GPC5-mutant LUAD patients exhibit altered tumor-infiltrating lymphocyte profiles and combine other *in vivo* and *in vitro* studies to explore the molecular mechanisms, thereby providing more in-depth therapeutic strategies. Additionally, combining β-catenin inhibitors with immune checkpoint blockade could be a rational strategy for this genetically defined subgroup, bridging genomic insights to immunotherapy optimization. Our discovery of a germline *GPC5* mutation associated with LUAD susceptibility highlights the importance of integrating germline genomics into precision oncology. Familial LUAD cases with *GPC5* mutations may require tailored surveillance strategies and early intervention with immunopreventive agents.

## Data Availability

The original contributions presented in the study are publicly available. This data can be found here: [https://www.ncbi.nlm.nih.gov/sra/PRJNA1240214].

## References

[B1] Alsaddah S. Papadakis A. I. Wong N. Palma L. Szlachtycz D. Cruz M. T. (2023). Germline EGFR c.2527G > A (p.V843I) variant and familial lung cancer. Lung Cancer 181, 107247. 10.1016/j.lungcan.2023.107247 37209596

[B2] Bao G. Guan X. Liang J. Yao Y. Xiang Y. Li T. (2022). A germline mutation in ATR is associated with lung adenocarcinoma in asian patients. Front. Oncol. 12, 855305. 10.3389/fonc.2022.855305 35712480 PMC9195140

[B3] Capurro M. Martin T. Shi W. Filmus J. (2014). Glypican-3 binds to Frizzled and plays a direct role in the stimulation of canonical Wnt signaling. J. Cell. Sci. 127 (Pt 7), 1565–1575. 10.1242/jcs.140871 24496449

[B4] Capurro M. I. Xiang Y. Y. Lobe C. Filmus J. (2005). Glypican-3 promotes the growth of hepatocellular carcinoma by stimulating canonical Wnt signaling. Cancer Res. 65 (14), 6245–6254. 10.1158/0008-5472.CAN-04-4244 16024626

[B5] Capurro M. I. Xu P. Shi W. Li F. Jia A. Filmus J. (2008). Glypican-3 inhibits Hedgehog signaling during development by competing with patched for Hedgehog binding. Dev. Cell. 14 (5), 700–711. 10.1016/j.devcel.2008.03.006 18477453

[B6] Chen N. Zhao S. Jolly A. Wang L. Pan H. Yuan J. (2021). Perturbations of genes essential for Müllerian duct and Wölffian duct development in Mayer-Rokitansky-Küster-Hauser syndrome. Am. J. Hum. Genet. 108 (2), 337–345. 10.1016/j.ajhg.2020.12.014 33434492 PMC7896104

[B7] Chui M. H. Have C. Hoang L. N. Shaw P. Lee C. H. Clarke B. A. (2018). Genomic profiling identifies *GPC5* amplification in association with sarcomatous transformation in a subset of uterine carcinosarcomas. J. Pathol. Clin. Res. 4 (1), 69–78. 10.1002/cjp2.89 29416878 PMC5783974

[B8] de Moraes G. F. A. Listik E. Justo G. Z. Vicente C. M. Toma L. (2021). The Glypican proteoglycans show intrinsic interactions with Wnt-3a in human prostate cancer cells that are not always associated with cascade activation. BMC Mol. Cell. Biol. 22 (1), 26. 10.1186/s12860-021-00361-x 33947326 PMC8097805

[B9] Eiamart W. Wonganan P. Tadtong S. Samee W. (2025). Panduratin A from boesenbergia rotunda effectively inhibits EGFR/STAT3/akt signaling pathways, inducing apoptosis in nsclc cells with wild-type and T790M mutations in EGFR. Int. J. Mol. Sci. 26 (5), 2350. 10.3390/ijms26052350 40076971 PMC11900324

[B10] Gabriel L. McVeigh T. Macmahon S. Avila Z. Donovan L. Hunt I. (2024). Familial rare EGFR-mutant lung cancer syndrome: review of literature and description of R776H family. Lung Cancer 191, 107543. 10.1016/j.lungcan.2024.107543 38569279

[B11] Ge D. Ma S. Sun T. Li Y. Wei J. Wang C. (2025). Pulmonary delivery of dual-targeted nanoparticles improves tumor accumulation and cancer cell targeting by restricting macrophage interception in orthotopic lung tumors. Biomaterials 315, 122955. 10.1016/j.biomaterials.2024.122955 39547139

[B12] He Q. Sun M. Sun N. Han Q. Shen Y. Li L. (2025). Polysocial risk score, lifestyle, genetic factors and risk of incident lung cancer. Public Health 242, 50–57. 10.1016/j.puhe.2025.02.024 40024208

[B13] Hemminki K. Försti A. Hemminki O. Scott R. J. Hemminki A. (2025). Age-specific familial risks in cancer as clues to germline genetic and environmental causes: focus on colorectal, endometrial, prostate, kidney, breast and lung cancers. Hered. Cancer Clin. Pract. 23 (1), 7. 10.1186/s13053-024-00301-8 39985094 PMC11844152

[B14] Hong X. Zhang Z. Pan L. Ma W. Zhai X. Gu C. (2019). MicroRNA-301b promotes the proliferation and invasion of glioma cells through enhancing activation of Wnt/β-catenin signaling via targeting Glypican-5. Eur. J. Pharmacol. 854, 39–47. 10.1016/j.ejphar.2019.03.057 30951720

[B15] Kiely M. Tse L. A. Koka H. Wang D. Lee P. Wang F. (2021). Age-related DNA methylation in paired normal and tumour breast tissue in Chinese breast cancer patients. Epigenetics 16 (6), 677–691. 10.1080/15592294.2020.1819661 32970968 PMC8143246

[B16] Landi M. T. Chatterjee N. Caporaso N. E. Rotunno M. Albanes D. Thun M. (2010). *GPC5* rs2352028 variant and risk of lung cancer in never smokers. Lancet Oncol. 11 (8), 714–716. 10.1016/S1470-2045(10)70096-6 PMC3238679 20688270

[B17] Li F. Shi W. Capurro M. Filmus J. (2011). Glypican-5 stimulates rhabdomyosarcoma cell proliferation by activating Hedgehog signaling. J. Cell. Biol. 192 (4), 691–704. 10.1083/jcb.201008087 21339334 PMC3044117

[B18] Liu J. Q. Liao X. W. Wang X. K. Yang C. K. Zhou X. Liu Z. Q. (2020). Prognostic value of Glypican family genes in early-stage pancreatic ductal adenocarcinoma after pancreaticoduodenectomy and possible mechanisms. BMC Gastroenterol. 20 (1), 415. 10.1186/s12876-020-01560-0 33302876 PMC7731467

[B19] Luo G. Zhang Y. Rumgay H. Morgan E. Langselius O. Vignat J. (2025). Estimated worldwide variation and trends in incidence of lung cancer by histological subtype in 2022 and over time: a population-based study. Lancet Respir. Med. 13, 348–363. 10.1016/S2213-2600(24)00428-4 39914442

[B20] Mukherjee A. Ye Y. Wiener H. W. Kuniholm M. H. Minkoff H. Michel K. (2023). Variations in genes encoding human papillomavirus binding receptors and susceptibility to cervical precancer. Cancer Epidemiol. Biomarkers Prev. 32 (9), 1190–1197. 10.1158/1055-9965.EPI-23-0300 37410084 PMC10472094

[B21] Müller P. Velazquez Camacho O. Yazbeck A. M. Wölwer C. Zhai W. Schumacher J. (2023). Why loss of Y? A pan-cancer genome analysis of tumors with loss of Y chromosome. Comput. Struct. Biotechnol. J. 21, 1573–1583. 10.1016/j.csbj.2023.02.024 36874157 PMC9978323

[B22] Niu F. Liu Y. Jing Z. Han G. Sun L. Yan L. (2018). Novel carbazole sulfonamide microtubule-destabilizing agents exert potent antitumor activity against esophageal squamous cell carcinoma. Cancer Lett. 420, 60–71. 10.1016/j.canlet.2018.01.066 29408653

[B23] Saez-Ibañez A. R. Upadhaya S. Partridge T. Shah M. Correa D. Campbell J. (2022). Landscape of cancer cell therapies: trends and real-world data. Nat. Rev. Drug Discov. 21 (9), 631–632. 10.1038/d41573-022-00095-1 35650421

[B24] Sung H. Ferlay J. Siegel R. L. Laversanne M. Soerjomataram I. Jemal A. (2021). Global cancer statistics 2020: GLOBOCAN estimates of incidence and mortality worldwide for 36 cancers in 185 countries. CA a cancer J. Clin. 71 (3), 209–249. 10.3322/caac.21660 33538338

[B25] Tan A. C. Saw S. P. L. Chen J. Lai G. G. Y. Oo H. N. Takano A. (2022). Clinical and genomic features of HER2 exon 20 insertion mutations and characterization of HER2 expression by immunohistochemistry in east asian non-small-cell lung cancer. JCO Precis. Oncol. 6, e2200278. 10.1200/PO.22.00278 36240473

[B26] Veugelers M. Vermeesch J. Reekmans G. Steinfeld R. Marynen P. David G. (1997). Characterization of glypican-5 and chromosomal localization of human *GPC5*, a new member of the glypican gene family. Genomics 40 (1), 24–30. 10.1006/geno.1996.4518 9070915

[B27] Wang K. Zhao S. Xie Z. Zhang M. Zhao H. Cheng X. (2022). Exome-wide analysis of *de novo* and rare genetic variants in patients with brain arteriovenous malformation. Neurology 98 (16), e1670–e1678. 10.1212/WNL.0000000000200114 35228337

[B28] Wang S. Qiu M. Xia W. Xu Y. Mao Q. Wang J. (2016). Glypican-5 suppresses Epithelial-Mesenchymal Transition of the lung adenocarcinoma by competitively binding to Wnt3a. Oncotarget 7 (48), 79736–79746. 10.18632/oncotarget.12945 27806326 PMC5346747

[B29] Xu F. Xiao C. Sun W. He Y. Chalela R. Masuda K. (2022). A lung adenocarcinoma patient with ROS1 fusion and NBN germline mutation achieves long progression-free survival from sintilimab combined with niraparib after failure of ROS1 inhibitors: a case report. Ann. Transl. Med. 10 (16), 912. 10.21037/atm-22-3582 36111030 PMC9469170

[B30] Yang H. Wang L. (2023). Heparan sulfate proteoglycans in cancer: pathogenesis and therapeutic potential. Adv. cancer Res. 157, 251–291. 10.1016/bs.acr.2022.08.001 36725112 PMC12276945

[B31] Yang X. Chen Y. Zhou Y. Wu C. Li Q. Wu J. (2021). *GPC5* suppresses lung cancer progression and metastasis via intracellular CTDSP1/AhR/ARNT signaling axis and extracellular exosome secretion. Oncogene 40 (25), 4307–4323. 10.1038/s41388-021-01837-y 34079082

[B32] Yang X. Zhang Z. Qiu M. Hu J. Fan X. Wang J. (2013). Glypican-5 is a novel metastasis suppressor gene in non-small cell lung cancer. Cancer Lett. 341 (2), 265–273. 10.1016/j.canlet.2013.08.020 23962560

[B33] Yu W. Inoue J. Imoto I. Matsuo Y. Karpas A. Inazawa J. (2003). *GPC5* is a possible target for the 13q31-q32 amplification detected in lymphoma cell lines. J. Hum. Genet. 48 (6), 331–335. 10.1007/s10038-003-0026-2 12721791

[B34] Zhang C. Gao L. Zhang Y. Jin X. Wang M. Wang Q. (2024). Corosolic acid inhibits EMT in lung cancer cells by promoting YAP-mediated ferroptosis. Phytomedicine Int. J. phytotherapy Phytopharm. 135, 156110. 10.1016/j.phymed.2024.156110 39369568

[B35] Zhang T. Joubert P. Ansari-Pour N. Zhao W. Hoang P. H. Lokanga R. (2021). Genomic and evolutionary classification of lung cancer in never smokers. Nat. Genet. 53 (9), 1348–1359. 10.1038/s41588-021-00920-0 34493867 PMC8432745

[B36] Zhao S. Zhang Y. Chen W. Li W. Wang S. Wang L. (2021). Diagnostic yield and clinical impact of exome sequencing in early-onset scoliosis (EOS). J. Med. Genet. 58 (1), 41–47. 10.1136/jmedgenet-2019-106823 32381727 PMC7802082

[B37] Zhi R. Li Q. Zhang H. Fan F. (2025). VPS45 contributes to the progression of hepatocellular carcinoma by triggering the wnt/β-catenin signaling pathway. Mol. Carcinog. 64 (4), 744–755. 10.1002/mc.23884 39835603 PMC11890426

[B38] Zhou Z. Li X. Yang G. Wang J. Li B. Huang Y. (2023). Targeting β-catenin and PD-L1 simultaneously by a racemic supramolecular peptide for the potent immunotherapy of hepatocellular carcinoma. Theranostics 13 (10), 3371–3386. 10.7150/thno.83377 37351175 PMC10283047

