# Supplementary figures and images for: A rare germline mutation reverses the suppressive effect of GPC5 thereby promoting lung adenocarcinoma development and tumorigenesis
Source: Front Genet. 2025 Apr 25;16:1582504. doi: 10.3389/fgene.2025.1582504 (PMC12062016; doi:10.3389/fgene.2025.1582504)

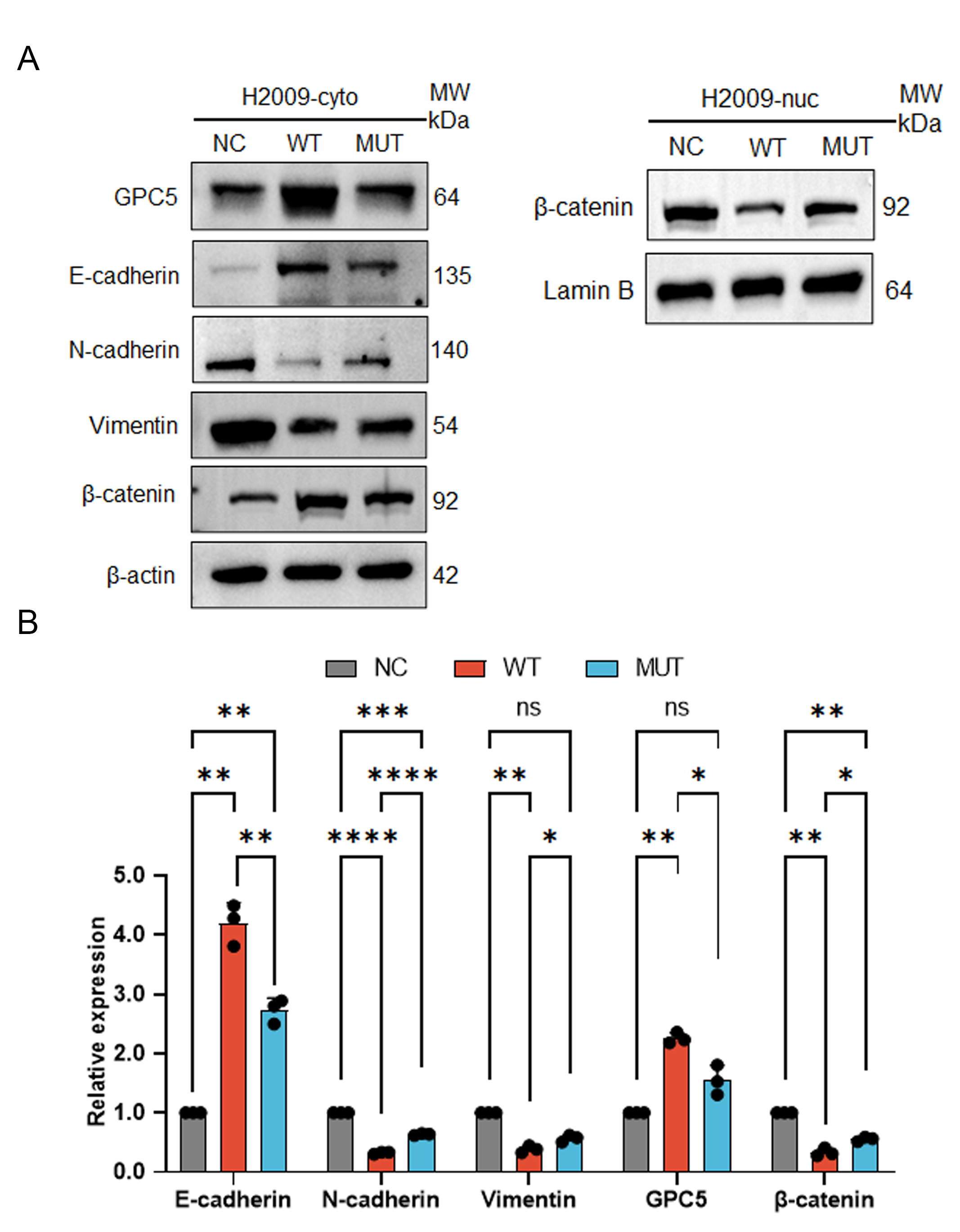

Supplement: Supplementary file 3 [file Image3.tif]

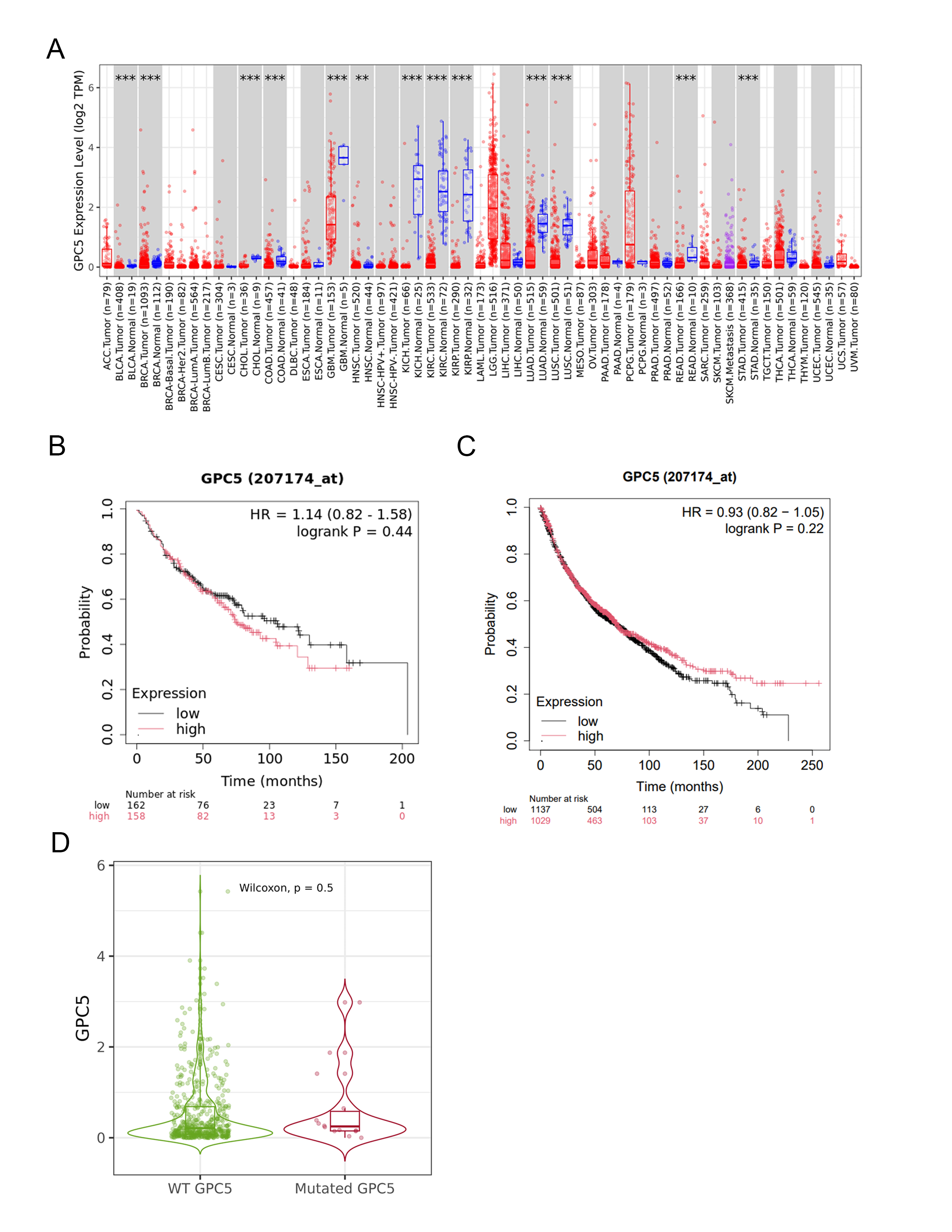

Supplement: Supplementary file 4 [file Image4.tif]

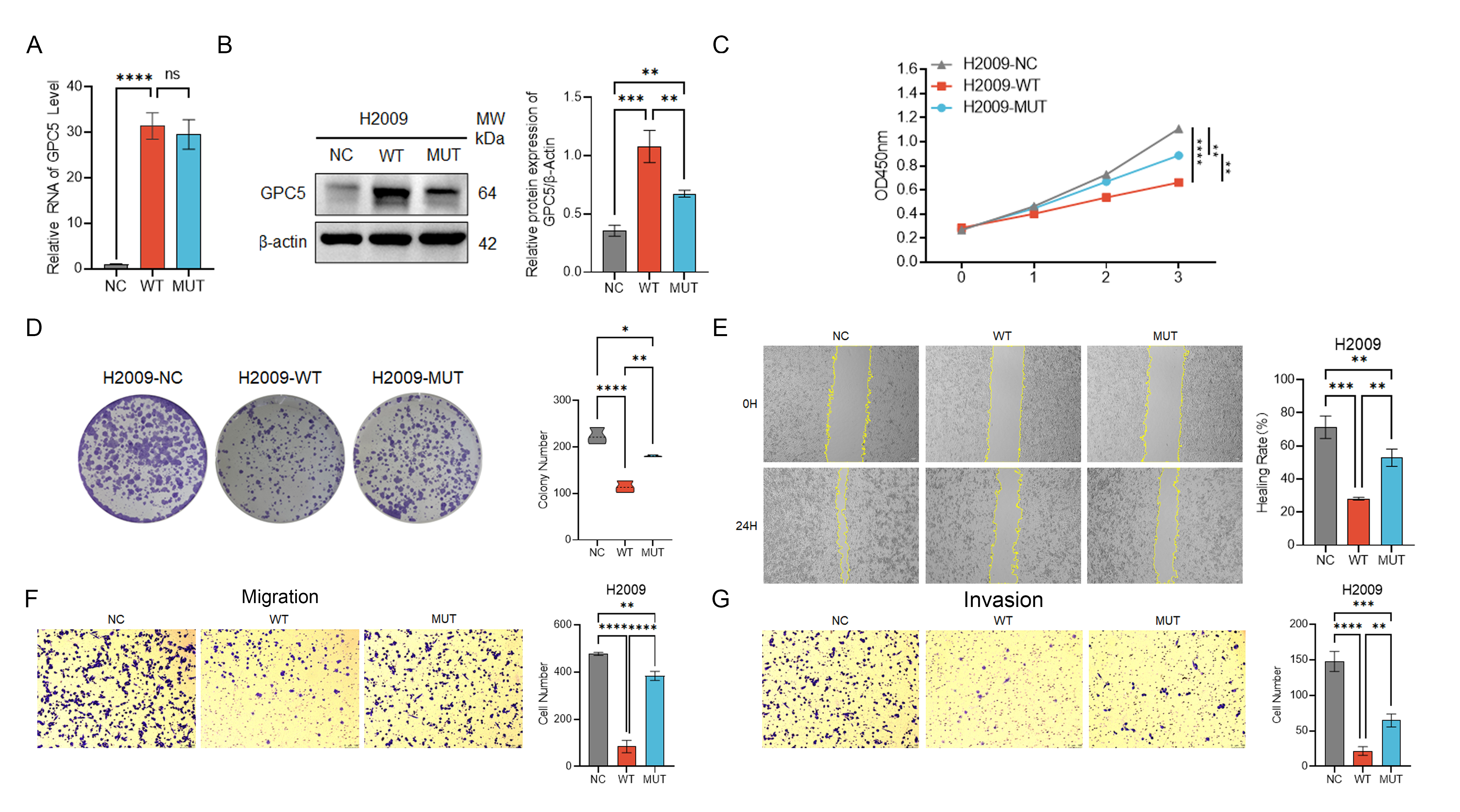

Supplement: Supplementary file 5 [file Image2.tif]

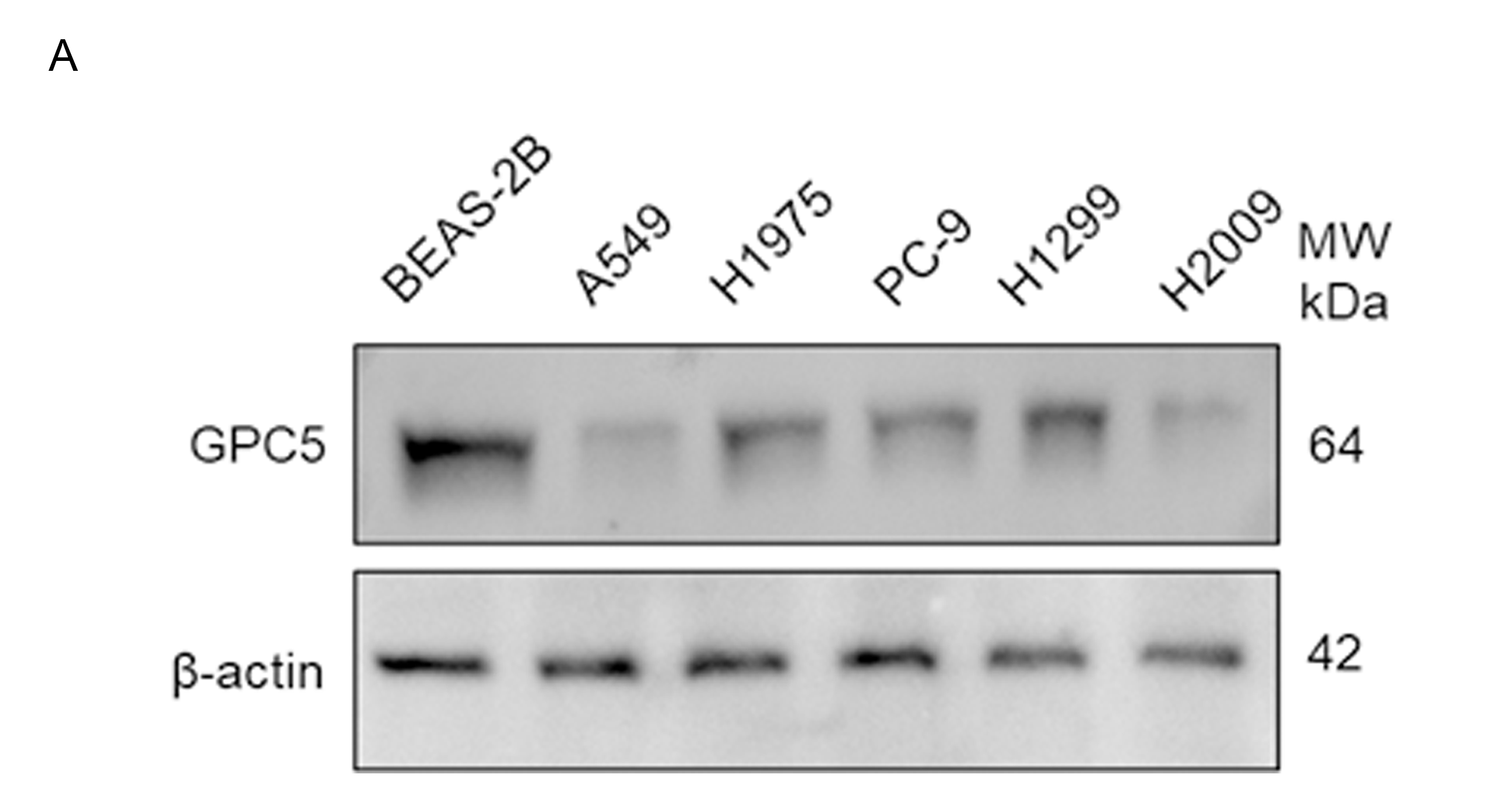

Supplement: Supplementary file 6 [file Image1.tif]
